# Direct Regulation of Aromatase B Expression by 17β-Estradiol and Dopamine D1 Receptor Agonist in Adult Radial Glial Cells

**DOI:** 10.3389/fnins.2015.00504

**Published:** 2016-01-12

**Authors:** Lei Xing, Crystal Esau, Vance L. Trudeau

**Affiliations:** Department of Biology, Centre for Advanced Research in Environmental Genomics, University of OttawaOttawa, ON, Canada

**Keywords:** 17β-estradiol, dopamine, aromatase, radial glial cell, teleost fish

## Abstract

Aromatase cytochrome P450arom (*cyp19*) is the only enzyme that has the ability to convert androgens into estrogens. Estrogens, which are produced locally in the vertebrate brain play many fundamental roles in neuroendocrine functions, reproductive functions, socio-sexual behaviors, and neurogenesis. Radial glial cells (RGCs) are neuronal progenitor cells that are abundant in fish brains and are the exclusive site of aromatase B expression and neuroestrogen synthesis. Using a novel *in vitro* RGC culture preparation we studied the regulation of aromatase B by 17β-estradiol (E2) and dopamine (DA). We have established that activation of the dopamine D1 receptor (D1R) by SKF 38393 up-regulates aromatase B gene expression most likely through the phosphorylation of cyclic AMP response element binding protein (CREB). This up-regulation can be enhanced by low concentration of E2 (100 nM) through increasing the expression of D1R and the level of p-CREB protein. However, a high concentration of E2 (1 μM) and D1R agonist together failed to up-regulate aromatase B, potentially due to attenuation of *esr2b* expression and p-CREB levels. Furthermore, we found the up-regulation of aromatase B by E2 and DA both requires the involvement of *esr1* and *esr2a*. The combined effect of E2 and DA agonist indicates that aromatase B in the adult teleost brain is under tight control by both steroids and neurotransmitters to precisely regulate neuroestrogen levels.

## Introduction

Aromatase cytochrome P450arom (*cyp19*) is the only enzyme performing the conversion of androgens into estrogens (Lephart, 1996; Garcia-Segura et al., 2003). In all species of vertebrates, including mammals, birds, and teleost fish, aromatase expression can be found in the brain in addition to testes and ovaries (Balthazart and Ball, 1998; Forlano et al., 2001). The distribution of aromatase varies between species and regions in the central nervous system (CNS), which has been detected at multiple levels, including mRNA, protein, and enzyme activity (Lephart, 1996; Balthazart and Ball, 1998; Forlano et al., 2006; Garcia-Segura, 2008; Azcoitia et al., 2011). In mammals, aromatase is expressed in both neuronal and glial cell bodies, processes and synaptic terminals of specific areas, especially the pial surface and ventricular surface in the brain under normal conditions (Martínez-Cerdeno et al., 2006; Yague et al., 2006). Aromatase may also be expressed in reactive astrocytes under conditions of cellular stress in mammalian brains (Azcoitia et al., 2003). In contrast to mammals, teleosts express two structurally and functionally different aromatase genes, aromatase A and aromatase B, which are a result of a gene duplication event (Diotel et al., 2010). The expression of aromatase B (*cyp19a1b*) in the teleost brain is exclusive to one particular cell type, the radial glial cell (RGC) (Forlano et al., 2001; Diotel et al., 2010; Xing et al., 2014), which allows direct *in vivo* study of glial aromatase contributions without interference from neuronal compartments. Callard et al. demonstrated for the first time that goldfish exhibit the highest brain aromatase activity compared to other vertebrates (Callard et al., 1981). More recently, Diotel et al. have documented the distribution *cyp11a*1, *3*β*-hsd, cyp17*, and *cyp19a1b* mRNAs and the ability of *de novo* synthesis of several steroids including estrone and 17β-estradiol (E2) in the adult zebrafish brain (Diotel et al., 2011). Although it is very difficult to compare aromatase activity among species, it is clear that activity in the forebrain of teleost fish is much higher (100–1000 times) than in birds and mammals. All these data indicate that teleost fish are excellent models to study function and regulation of glial aromatase. Better understanding the regulation of glial aromatase will widen our knowledge of the control of estrogens in the brain. Local formation of estrogens are extremely important to maintain the homeostasis of cell proliferation and cell death, since estrogens in the brain are proven to be neuroprotective in mammalian brains and contributing to the generation of new neurons and brain repair after injury (Azcoitia et al., 2003; Arevalo et al., 2015). More importantly, recent studies indicate that RGCs are the progenitors of both neurons and glia during development, and are the source of new neurons in adult vertebrate brains (Alvarez-Buylla et al., 2001; Zupanc and Clint, 2003; Pellegrini et al., 2007). All these lines of evidence suggest that both RGC progenitor and steroidogenic functions contribute to neurogenesis in adult vertebrate brains.

Brain aromatization plays numerous important roles including brain differentiation, neural plasticity, neural regeneration, neuroendocrine functions, reproductive functions, and socio-sexual behaviors. It is possible that these processes are in part regulated by aromatization of androgen and thus local estrogens formation (Lephart, 1996; Garcia-Segura et al., 1999, 2003; Azcoitia et al., 2003; Ubuka et al., 2014). There are many response elements located at the promoter region of teleost *cyp19a1b* (Callard et al., 2001; Tchoudakova et al., 2001), including estrogen response element (ERE) and cyclic adenosine monophosphate (cAMP) response element (CRE). Through differential response element recruitment, *cyp19a1b* expression can be regulated differently, which suggests there should be multiple factors implicated in the regulation of *cyp19a1b* expression (Kato et al., 1997). It is well known that estrogens can up-regulate *cyp19a1b* expression in fish brain, which is mediated by estrogen receptors (ERs), some of which act as ligand-activated transcription factors (Belcher and Zsarnovszky, 2001). Upon binding to classical nuclear ERs, activated ER homo-dimerizes, and binds to ERE at the promoter of *cyp19a1b*, and then mediates transcription (Marlatt et al., 2008; Strobl-Mazzulla et al., 2008). Due to duplication of the estrogen receptor beta gene, teleost have three ERs (*esr1*/ERα, *esr2a*/ERβ, and *esr2b*/ERγ), all of which are expressed in the brain of fish with an expression pattern similar to that of aromatase (Forlano et al., 2005; Pellegrini et al., 2005; Strobl-Mazzulla et al., 2008; Fergus and Bass, 2013). There is evidence showing that moderate up-regulation of ERα by estrogens precedes the dramatic up-regulation of aromatase by its own products (Denslow et al., 2001; Bowman et al., 2002; McEwen, 2002; Marlatt et al., 2008). The possibility that *cyp19a1b* expression is up-regulated by estrogens is intriguing and suggests the presence of a positive feedback loop within the aromatase and ER-positive cells, whereby estrogens enhance aromatase expression and therefore the subsequent products, estrogens.

In addition to estrogenic regulation of the *cyp19a1b* gene, our previous neuroanatomical and pharmacological studies in goldfish reveal that there is a close relationship between catecholaminergic neurons and aromatase B- and DA D1 receptor (D1R)-positive RGCs in goldfish forebrain (Xing et al., 2015). Activation of D1R but not D2R up-regulates *cyp19a1b* mRNA in cultured RGCs (Xing et al., 2015). The signaling pathway underlying activated D1R regulation of *cyp19a1b* expression involves cAMP, protein kinase A (PKA) and phosphorylated cAMP response element binding protein (p-CREB). Whether this regulation involves nuclear ERs has yet to be characterized. Since it is likely that both mechanisms are acting simultaneously to affect multifactorial control over neuroestrogen synthesis, and given the close association between RGCs and DA neurons we directly tested for interactive effects on RGCs *in vitro*.

In this report, we show that both E2 and D1R agonist SKF 38393 alone significantly increased the expression of *cyp19a1b*. Moreover, the expression of *cyp19a1b* in RGCs may be synergistically up-regulated or antagonistically down-regulated, respectively, under low-concentration or high-concentration exposure to E2 and SKF 38393.

## Materials and methods

### Ethics statement

All procedures used were approved by the University of Ottawa Protocol Review Committee and followed standard Canadian Council on Animal Care guidelines on the use of animals in research.

### Cell culture

Common adult female goldfish (*Carassius auratus*) were purchased in April from a commercial supplier (Mt. Parnell Fisheries Inc., Mercersburg, PA, USA) and acclimated for at least 3 weeks prior to experimentation. The fish were maintained at 18°C under a natural simulated photoperiod and fed standard goldfish flakes. Sexually mature, pre-spawning female goldfish (20–35 g) were anesthetized using 3-aminobenzoic acid ethyl ester (MS222) for all handling, injection, and dissection procedures.

Cell culture methods have been established and validated previously by Xing et al. (2015). Briefly, female goldfish hypothalamus and telencephalon were dissected and rinsed with Hanks Balanced Salt Solution (HBSS; 400 mg KCl, 600 mg KH_2_PO4, 350 mg NaHCO_3_, 8 g NaCl, 48 mg Na_2_HPO_4_, and 1 g D-Glucose in 1 L ddH_2_O) with Antibiotic-Antimycotic solution (Gibco) and minced into small explants. Radial glial cells were dissociated with trypsin (0.25%, Gibco) and cultured in Leibovitz's L-15 medium with 15% Fetal Bovine Serum (FBS) and Antibiotic-Antimycotic solution. Cell culture medium was changed 4–7 days after isolation to remove tissue explants and suspended cells. Following the initial media change, cells received fresh media once a week until they were ready for their next passage. RGCs were sub-cultured following trypsinization (0.125%) for three passages and then used for experiments.

### Drugs and exposures

Cells were exposed to E2 (Sigma-Aldrich) and selective DA D1R agonist SKF 38393 (Tocris) for 24 h to study their effects on *cyp19a1b, esr1, esr2a, esr2b*, and *drd1a* mRNA and p-CREB protein level. To investigate ERs involvement in *cyp19a1b* mRNA regulation by E2 and SKF 38393, cells were pre-exposed to estrogen receptor antagonist MPP (Tocris) for 1 h, and then exposed to E2 or SKF 38393 for 24 h.

### RNA extraction, quality control and cDNA synthesis

RNA was isolated using the RNeasy Micro kit (Qiagen) as described in the manufacturer's protocol. Upon purification, concentration and quality of all samples were assessed using the NanoDrop ND-1000 spectrophotometer (Thermo Scientific) and the 2100 Bioanalyzer (Agilent). RNA integrity values of samples were above the recommended minimum value of 5 for quantitative real-time RT PCR applications and ranged from 8.5 to 9.8 (Fleige and Pfaffl, 2006). Total cDNA was prepared using Maxima First Strand cDNA Synthesis Kit for RT-qPCR (Thermo Scientific). Each 20 μl reaction was diluted 10-fold in nuclease-free water and used as the template for the real-time RT-PCR assays.

### Quantitative real-time RT-PCR

Quantitative real-time RT-PCR (qPCR) assays based on SYBR green detection were used to validate relative gene expression. Primers used in this study were designed using the Primer3 (http://primer3.sourceforge.net/) software and synthesized by Invitrogen (Table 1). The Maxima SYBR green qPCR Master Mix (Thermo Scientific) and CFX96 Real-Time PCR Detection System (Bio-Rad) were used to amplify and detect the transcripts of interest. Thermal cycling conditions included a denaturation step at 95°C for 3 min, and then 40 cycles of denaturation at 95°C for 10 s, annealing at 60°C for 30 s followed by melt curve analysis to confirm product specificity for all transcripts. Serial 1/2 dilution of the pool of all cDNA samples was analyzed to build the standard curves, from which the mRNA abundance in samples was calculated using the CFX Manager™ Software package (Bio-Rad). The efficiencies for all standard curves were between 92 and 106%. Data were normalized by using NORMA-GENE algorithm (Heckmann et al., 2011) and then presented as means + SEM of fold change to control group (*n* = 4; assayed in duplicate).

**Table 1:** **Primer sets used for qPCR and ddPCR**.

**Gene**	**Primer sequence (Forward)**	**Primer sequence (Reverse)**	**Amplicon size**	**Accession no**.
*β-actin*	CTGGGATGATATGGAGAAGA	CCAGTAGTACGACCTGAAGC	215	AB039726
*18s*	AAACGGCTACCACATCCAAG	CACCAGATTTGCCCTCCA	165	AF047349
*cyp19a1b*	TGCTGACATAAGGGCAATGA	GGAAGTAAAATGGGTTGTGGA	153	AB009335
*esr1*	GCAGGAGGGTTTGATTCTGAGA	CCATAATGATAGCCGGACGCA	76	AY055725
*esr2a*	TGACTACATCTGCCCTGCCA	CCAACTTCGTAACATTTTCGGAGA	72	AF061269
*esr2b*	TGGTCCCTTTAAATTCAGCAATCT	GTGTTTCCCTGTAGGCCAGTG	95	AF177465
*drd1a*	CCCTTTGGTGCGTTTTGT	GAGCCTTGTGCCATTTGAG	210	L08602

### Droplet digital PCR

The low expression of D1R (*drd1a*) in RGC cultures is not quantifiable using qPCR, so we used a more sensitive method to measure *drd1a* mRNA level. Droplet digital PCR (ddPCR) was performed using 25 ng of each cDNA sample and QX200™ ddPCR™ EvaGreen Supermix by employing QX200™ Droplet Digital™ PCR System (Bio-Rad). The ddPCR conditions were as follows: an initial denaturation for 5 min at 95°C followed by 40 cycles of denaturation for 30 s at 95°C and annealing and extension for 1 min at 56°C, then signal stabilization for 5 min at 4°C and 5 min at 90°C. Template DNA was omitted from the ddPCR reaction as a no template control (NTC) and the results of ddPCR were analyzed using the QuantaSoft Software (Bio-Rad). The absolute copy number of targeted gene was divided by the average of the absolute copy number of two reference genes β-actin and 18s, and presented as means + SEM of fold change to control group (*n* = 4; assayed in duplicate).

### Western blot

Total protein extract from RGCs was denatured and separated by electrophoresis on a 10% SDS–polyacrylamide gel and transferred to a PVDF membrane as described previously by Zhao et al. (2006). Membranes were incubated with anti-Phospho-CREB (Ser133, Cell signaling technology, 9198S) antibodies overnight at 4°C and then incubated with donkey anti-rabbit IgG antibody (GE Health Care, NA934VS). Blots were visualized using the Amersham ECL Prime Western Blotting Detection System (GE Health Care, RPN2232). Images were obtained using a ChemiDoc XRS+ system (Bio-Rad) and analyzed with image lab (Bio-Rad). Same blot incubated with anti-Phospho-CREB antibodies before was stripped and re-probed with primary anti-actin (Cedarlane, CLT9001) antibodies overnight at 4°C and then incubated with goat anti-mouse IgG-HRP (Santa Cruz, sc-2005), where actin served as an internal control.

### Statistics

In all cases and before any analysis, normality and homogeneity of variances was verified using Shapiro-Wilk's and Levene's test, respectively. Comparison of two groups was performed using Student's *t*-test in GraphPad Prism (Version 6.0). Two-way analysis of variance (ANOVA) followed by Tukey's *post-hoc* test in GraphPad Prism (Version 6.0) was used to determine the effects of single or combined drug treatments. *P* = 0.05 were considered statistically significant. Data were presented as mean + SEM. Note that the *t*- and *F*-statistics, degrees of freedom (df) of interaction (DFn), the total degrees of freedom (DFd), and *P*-values are reported in the Results Section. The *P*-values associated with main effects and the interactions are reported directly on the graphs.

## Results

### Regulation of cyp19a1b and ER expression by SKF 38393 and E2

**Figure 1:**
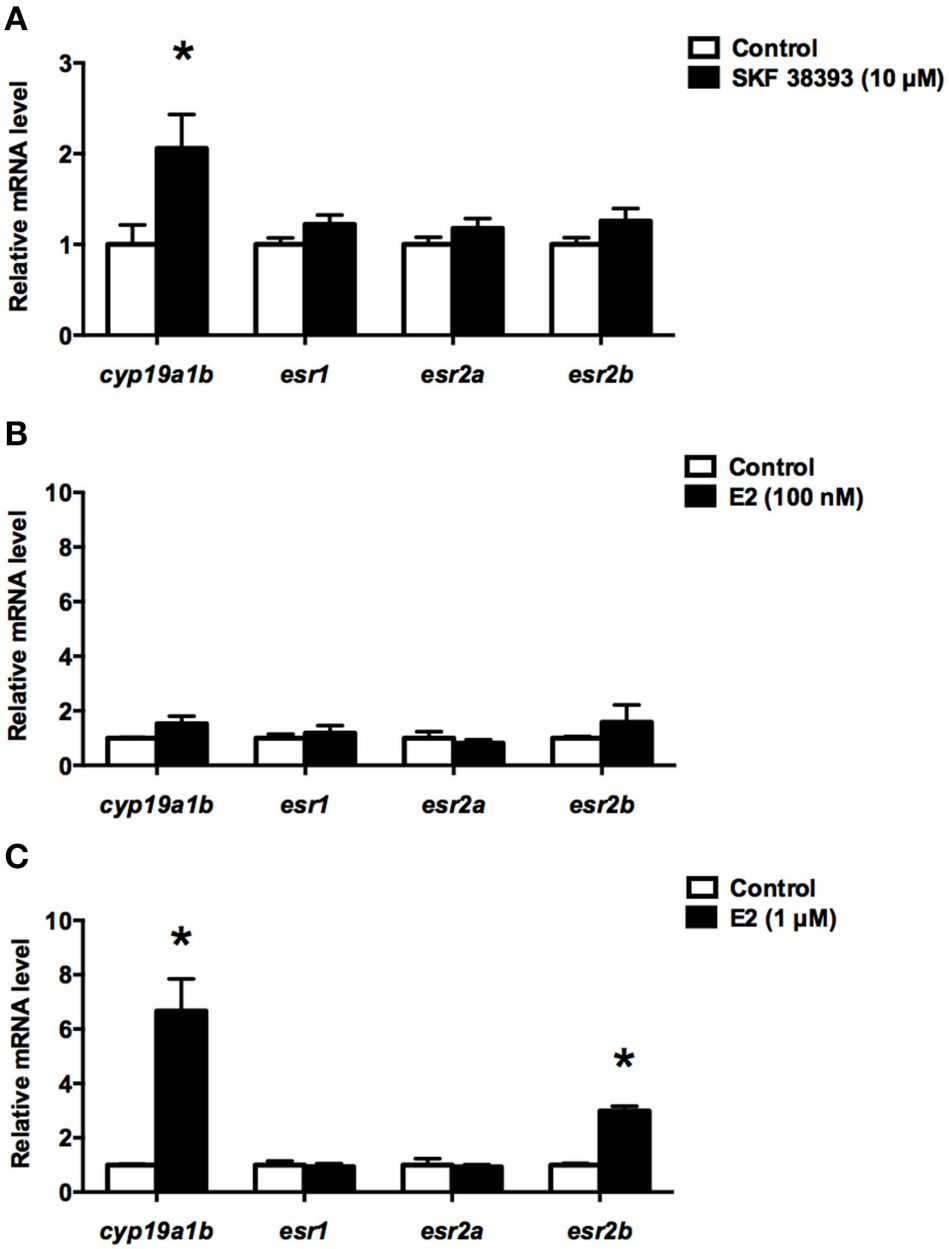
**Effects of SKF 38393 and E2 on the expression of ***cyp19a1b***, ***esr1, esr2a***, and ***esr2b*** in primary RGC culture**. Quantitative real-time PCR analysis showing variations in the relative amounts of the *cyp19a1b, esr1, esr2a*, and *esr2b* mRNAs in primary RGCs culture exposed to 10 μM SKF38393 **(A)** 100 nM E2 **(B)** and 1 μM E2 **(C)**. Data were normalized and defined as fold change relative to control, and bars represent the mean + SEM. Treatment groups marked by asterisks have significantly different mRNA levels compared to control (*P* < 0.05).

Here, the qPCR data show that 10 μM SKF 38393 was able to increase the expression of *cyp19a1b* (*t* = 2.454, df = 6, *P* = 0.0225), but not *esr1* (*t* = 1.744, df = 6, *P* = 0.0952)*, esr2a* (*t* = 1.338, df = 6, *P* = 0.1947), and *esr2b* (*t* = 1.638, df = 22, *P* = 0.1157) after 24 h incubation (Figure 1A). The 100 nM concentration of E2 did not affect *cyp19a1b* (*t* = 1.557, df = 6, *P* = 0.1705), *esr1* (*t* = 0.5747, df = 6, *P* = 0.5864), *esr2a* (*t* = 0.5106, df = 6, *P* = 0.6993), and *esr2b* (*t* = 0.9012, df = 6, *P* = 0.4022) mRNAs after 24 h incubation (Figure 1B). However, when the concentration of E2 was increased to 1 μM, the expression of *cyp19a1b* (*t* = 4.791, df = 6, *P* = 0.003) and *esr2b* (*t* = 10.65, df = 6, *P* < 0.0001) were increased dramatically 6.7-fold and 2.9-fold, respectively, but not *esr1* (*t* = 0.3295, df = 6, *P* = 0.7530) and *esr2a* (*t* = 0.2395, df = 6, *P* = 0.779; Figure 1C). These data suggest the up-regulation of *esr2b* may contribute to the stimulatory effect of E2 in *cyp19a1b* expression.

### The involvement of ERs in cyp19a1b regulation by E2 and SKF 38393

**Figure 2:**
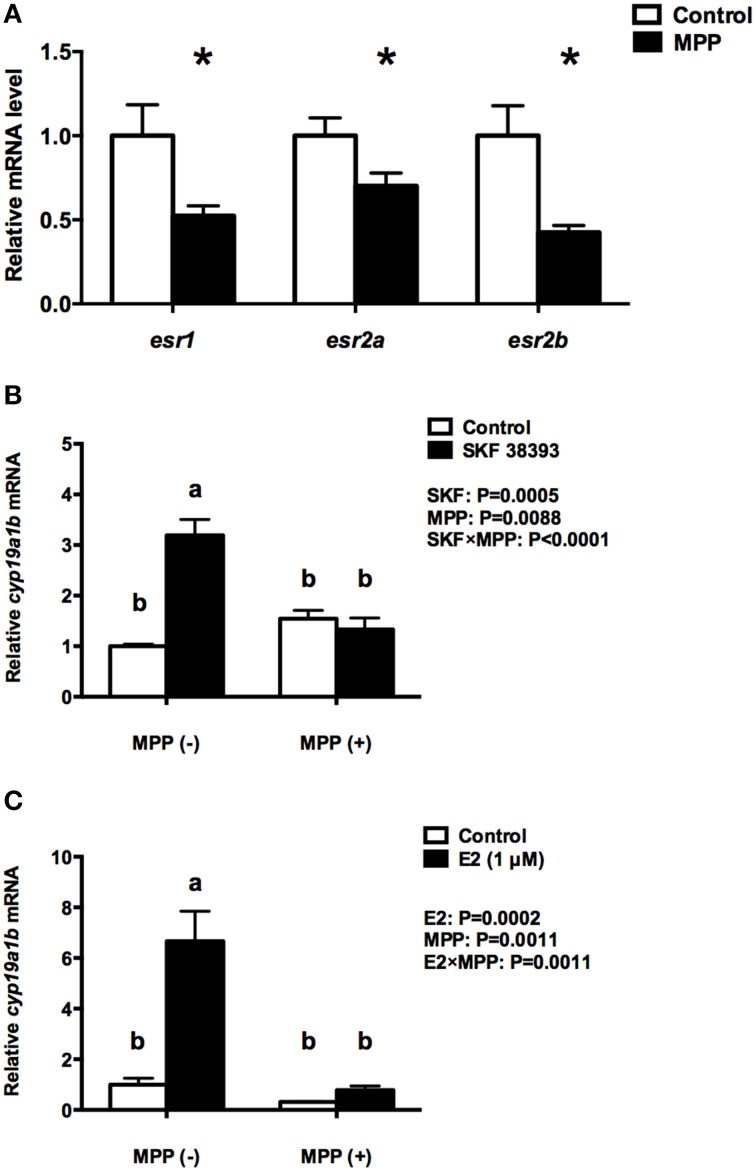
**Effects of MPP on the expression of ***esr1, esr2a***, and ***esr2b*** and the regulation of ***cyp19a1b*** expression by DA and E2 in primary RGC culture**. Quantitative real-time PCR analysis showing variations in the relative amounts of the *cyp19a1b, esr1, esr2a*, and *esr2b* mRNAs in primary RGCs culture exposed to 100 nM MPP **(A)**, 100 nM MPP and 10 μM SKF 38393 **(B)**, 100 nM MPP and 1 μM E2 **(C)**. Data were normalized and defined as fold change relative to control, and bars represent the mean + SEM. Treatment groups marked by an asterisk **(A)** are significantly different from controls (*P* < 0.05). Treatment groups marked with different letters (**B,C**) are significantly different (*P* < 0.05).

When RGC cultures are exposed to SKF38393 and E2 for 24 h there is no change in the expression of *esr1* and *esr2a*. This result led us to investigate the possible contribution of pre-existing ERs in RGCs to *cyp19a1b* up-regulation. The ERα and ERβ antagonist MPP was administered to RGC culture 1 h before SKF 38393 and E2. Data revealed that MPP alone decreases the expression of all three ERs (*t* = 2.462, df = 6, *P* = 0.049; *t* = 2.285, df = 6, *P* = 0.0624; *t* = 3.134, df = 6, *P* = 0.0202, respectively) by 29.9–57.6% (Figure 2A), but has no effect on *cyp19a1b* (*F* = 32.65, DFn = 1, DFd = 12, *P* = 0.8582; *F* = 18.15, DFn = 1, DFd = 12, *P* = 0.3065, respectively) expression (Figures 2B,C). Two-way ANOVAs were conducted that examined the effect of MPP and SKF 38393 as well as MPP and E2 on *cyp19a1b* expression. There was a statistically significant interaction between MPP and SKF 38393 (*F* = 32.65, DFn = 1, DFd = 12, *P* < 0.0001), and between MPP and E2 (*F* = 18.15, DFn = 1, DFd = 12, *P* = 0.0011). The stimulatory effect of SKF 38393 (*F* = 32.65, DFn = 1, DFd = 12, *P* = 0.0002) and E2 (*F* = 18.15, DFn = 1, DFd = 12, *P* < 0.0001) on *cyp19a1b* mRNA was completely blocked by MPP (Figures 2B,C).

### The combined effects of E2 and SKF 38393 on cyp19a1b regulation

**Figure 3:**
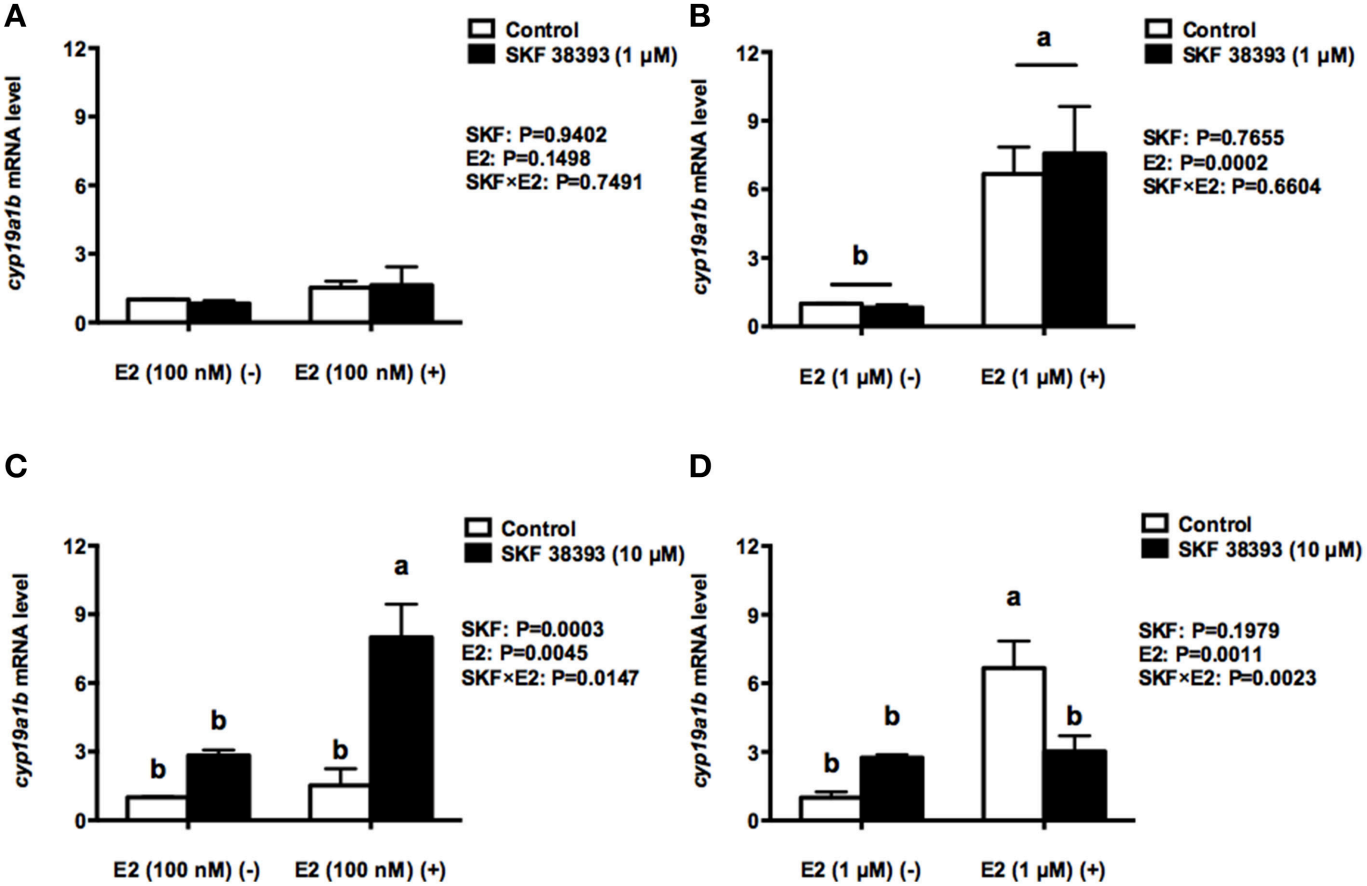
**Quantitative real-time PCR analysis showing variations in the relative amounts of the ***cyp19a1b*** mRNA in primary RGCs culture exposed to 100 nM E2 and 1 μM SKF 38393 (A) 1 μM E2 and 1 μM SKF 38393 (B) 100 nM E2 and 10 μM SKF 38393 (C) 1 μM E2 and 10 μM SKF 38393 (D)**. Data were normalized and defined as fold change relative to control, and bars represent the mean + SEM. Treatment groups marked by different letters are significantly different (*P* < 0.05).

Based on the previous dose-dependent studies (Xing et al., 2015 and unpublished data), we examined the combined effects of two concentrations of SKF 38393 and E2. Our results indicate that there is either a synergistic or antagonistic effect in *cyp19a1b* regulation by E2 and SKF 38393, which is dependent on the concentration of E2. Two-way ANOVAs were conducted that examined the effect of SKF 38393 and E2 on *cyp19a1b* expression. As shown in Figure 3A, the low concentration of E2 (100 nM) alone increased *cyp19a1b* expression 1.5 fold, but this was not statistically significant (*F* = 0.1071, DFn = 1, DFd = 12, *P* = 0.1498) regardless of the combination with SKF 38393 (1 μM). In contrast, the high concentration of E2 (1 μM) alone increased *cyp19a1b* 6.7-fold (*F* = 0.2028, DFn = 1, DFd = 12, *P* = 0.0002, Figure 3B) significantly regardless of the combination with SKF 38393 (1 μM). There was no statistically significant interaction between SKF 38393 (1 μM) and either the low (100 nM; *F* = 0.1071, DFn = 1, DFd = 12, *P* = 0.7491, Figure 3A) or higher (1 μM; *F* = 0.2028, DFn = 1, DFd = 12, *P* = 0.6601, Figure 3B) concentration of E2. When the concentration of SKF 38393 was increased to 10 μM, statistically significant interactions between SKF 38393 and both low concentration (*F* = 8.112, DFn = 1, DFd = 12, *P* = 0.0147, Figure 3C) and high concentration of E2 (*F* = 14.91, DFn = 1, DFd = 12, *P* = 0.0023, Figure 3D) were evident. The Two-way ANOVA indicates that both 10 μM SKF 38393 and 100 nM E2 increased *cyp19a1b* expression 2.8-fold and 1.5-fold, consistent with the earlier experiment shown in Figures 1A,B, respectively. *Post-hoc* tests showed that the combination of high concentration of SKF 38393 and low concentration of E2 synergistically up-regulated *cyp19a1b* (*F* = 8.112, DFn = 1, DFd = 12, *P* = 0.0003, Figure 3C) expression 8.0-fold. Contrasting this is the experiment where high concentration of SKF 38393 and high concentration of E2 were used. Exposure of RGCs to 1 μM E2 alone increased *cyp19a1b* expression 6.7-fold, and the high concentration of SKF 38393 inhibited this effect (*F* = 14.91, DFn = 1, DFd = 12, *P* = 0.0141, Figure 3D).

### Synergistic effects of low levels of E2 on SKF 38393-induced cyp19a1b expression are associated with parallel increases in p-CREB

**Figure 4:**
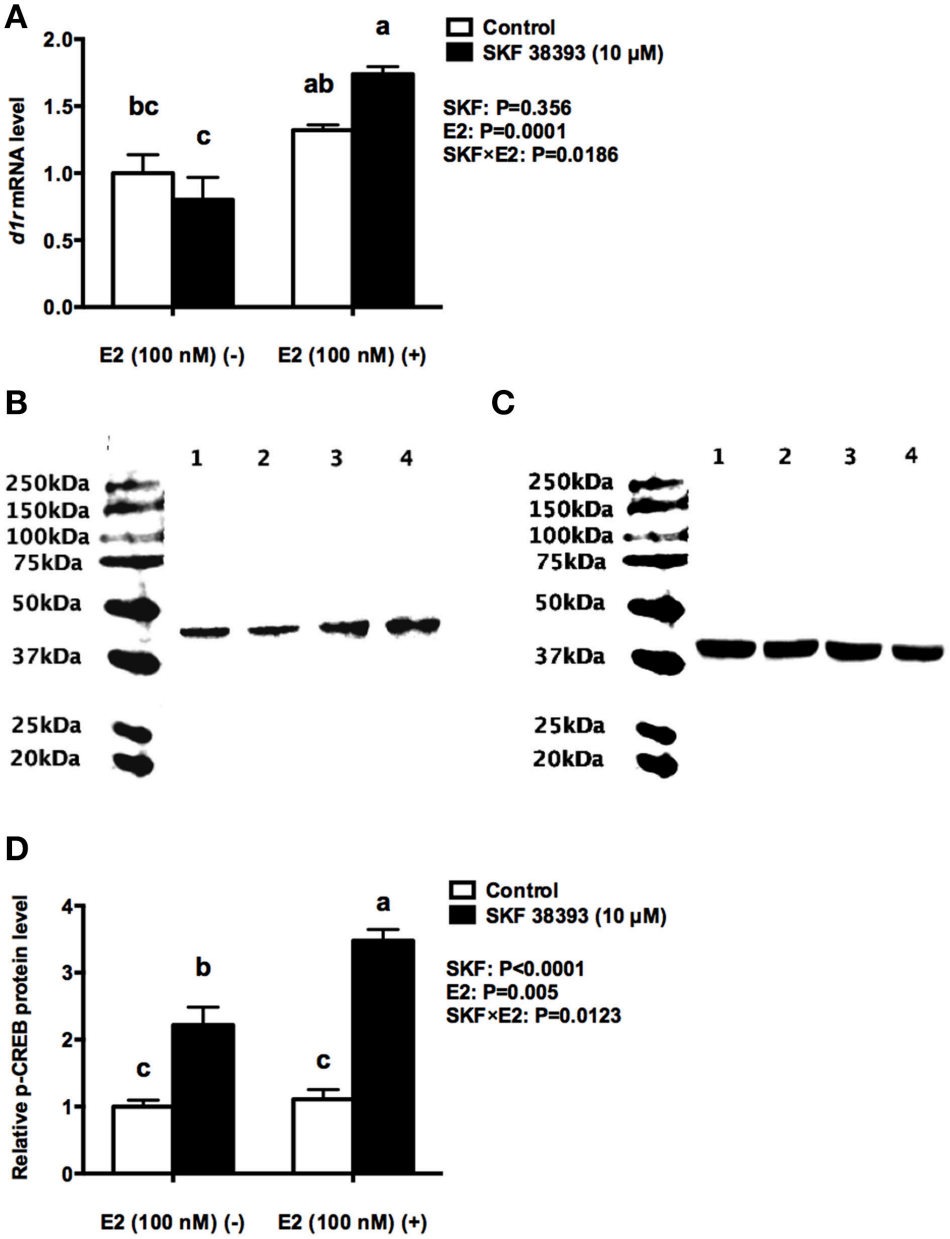
**Synergistic up-regulation of D1R and p-CREB by E2 and SKF 38393**. Droplet digital PCR analysis showing variations in the absolute amounts of the *drd1a* mRNA in primary RGCs culture exposed to 100 nM E2 and 10 μM SKF38393 **(A)**. Western blot showing variations in the relative protein level of p-CREB in primary RGCs culture exposed to 100 nM E2 and 10 μM SKF 38393. Western blot image **(B)** showing the p-CREB immunoreactivity in control (lane 1), 100 nM E2 treated (lane 2), 10 μM SKF 38393 treated (lane3), and both 100 nM and 10 μM SKF 38393 treated (lane 4) groups, where actin served as internal control **(C)**. The densitometric analysis of western blot is reported as an arbitrary value relative to the average of all bands on the same blot. Data were normalized and defined as fold change relative to control, and bars represent the mean + SEM (*n* = 4, **D**). Treatment groups marked by different letters are significantly different (*P* < 0.05).

**Figure 5:**
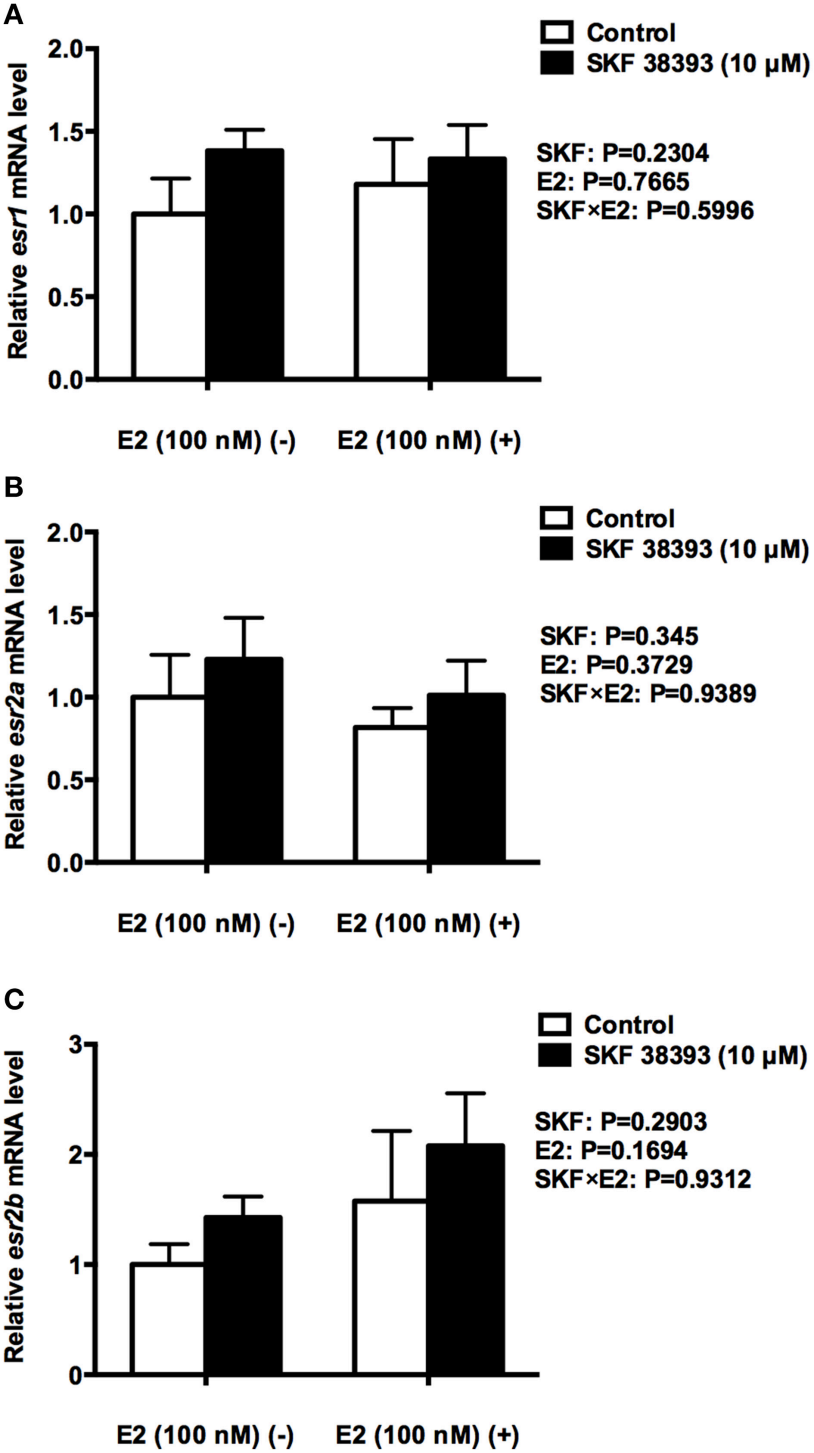
**Quantitative real-time PCR analysis showing variations in the relative amounts of the ***esr1*** (A), ***esr2a*** (B) and ***esr2b*** mRNA (C) in primary RGC cultures exposed to 100 nM E2 and 10 μM SKF 38393**. Data were normalized and defined as fold change relative to control, and bars represent the mean + SEM. There were no statistically signficant treatment effects.

To further understand the underlying mechanisms of synergistic up-regulation of *cyp19a1b* by low concentration of E2 and high concentration of SKF 38393, we studied two components of the D1R/cAMP/PKA/p-CREB pathway, *drd1a* in mRNA level and p-CREB in protein level as well as the expression of *esr1, esr2a*, and *esr2b*. Two-way ANOVAs were conducted that examined the effect of SKF 38393 and E2 on *drd1a, esr1 esr2a*, and *esr2b* mRNA level and p-CREB protein level in cultured RGCs. There was a statistically significant interaction (*F* = 7.393, DFn = 1, DFd = 12, *P* = 0.01786) between SKF 38393 and E2 for *drd1a* mRNA. *Post-hoc* tests showed that the combination of SKF 38393 and E2 up-regulated *drd1a* mRNA (*F* = 7.393, DFn = 1, DFd = 12, *P* = 0.0029; Figure 4A), although increases were not very large, in the range of 1.7 fold. There was also a statistically significant interaction (*F* = 10.35, DFn = 1, DFd = 12, *P* = 0.0123) between SKF 38393 and E2 for p-CREB protein level (Figures 4B–D). The D1R agonist SKF 38393 alone increased p-CREB 2.2-fold, and this was enhanced to 3.5-fold in the presence of E2, paralleling these results for *cyp19a1b* mRNA (Figure 3C). No effects on the expression of *esr1, esr2a* and *esr2b* (*F* = 0.2907, DFn = 1, DFd = 12, *P* = 0.5996; *F* = 0.0061, DFn = 1, DFd = 12, *P* = 0.9389; *F* = 0.0078, DFn = 1, DFd = 12, *P* = 0.9312, respectively) were observed for the E2 and SKF 38393 treatments (Figure 5).

### Antagonistic effects of high levels of E2 on SKF 38393-induced cyp19a1b expression are associated with parallel decreases in p-CREB protein and esr2b mRNA

**Figure 6:**
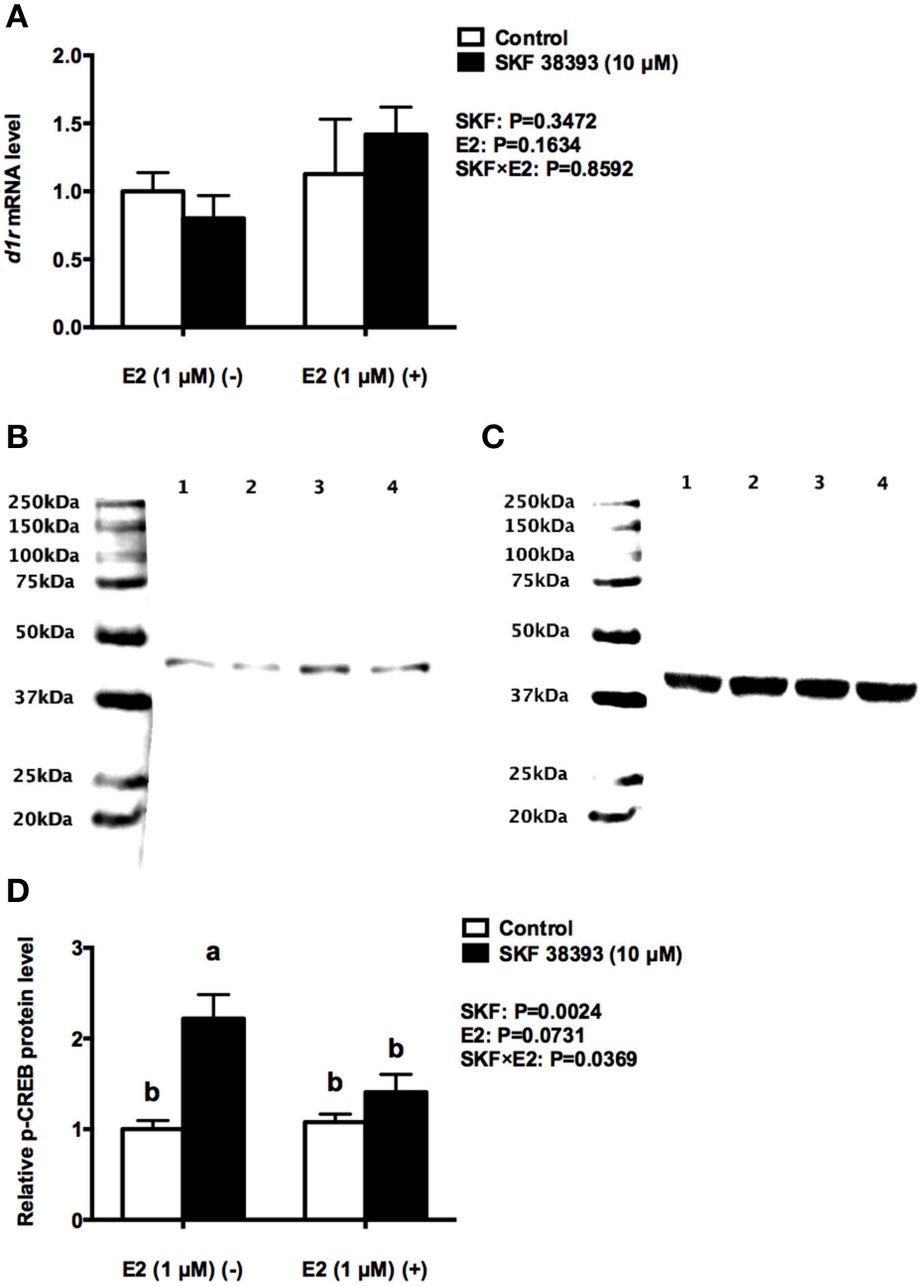
**Antagonistic regulation of D1R and p-CREB by E2 and SKF 38393**. Droplet digital PCR analysis showing variations in the absolute amounts of the *d1r* mRNA in primary RGCs culture exposed to 1 μM E2 and 10 μM SKF 38393 **(A)**. Western blot image **(B)** showing the p-CREB immunoreactivity in control (lane 1), 1 μM E2 treated (lane 2), 10 μM SKF 38393 treated (lane3), and both 1 μM and 10 μM SKF 38393 treated (lane 4) groups, where actin served as internal control **(C)**. The densitometric analysis of western blot is reported as an arbitrary value relative to the average of all bands on the same blot. Data were normalized and defined as fold change relative to control, and bars represent the mean + SEM (*n* = 4, **D**). Treatment groups marked by different letters are significantly different (*P* < 0.05).

**Figure 7:**
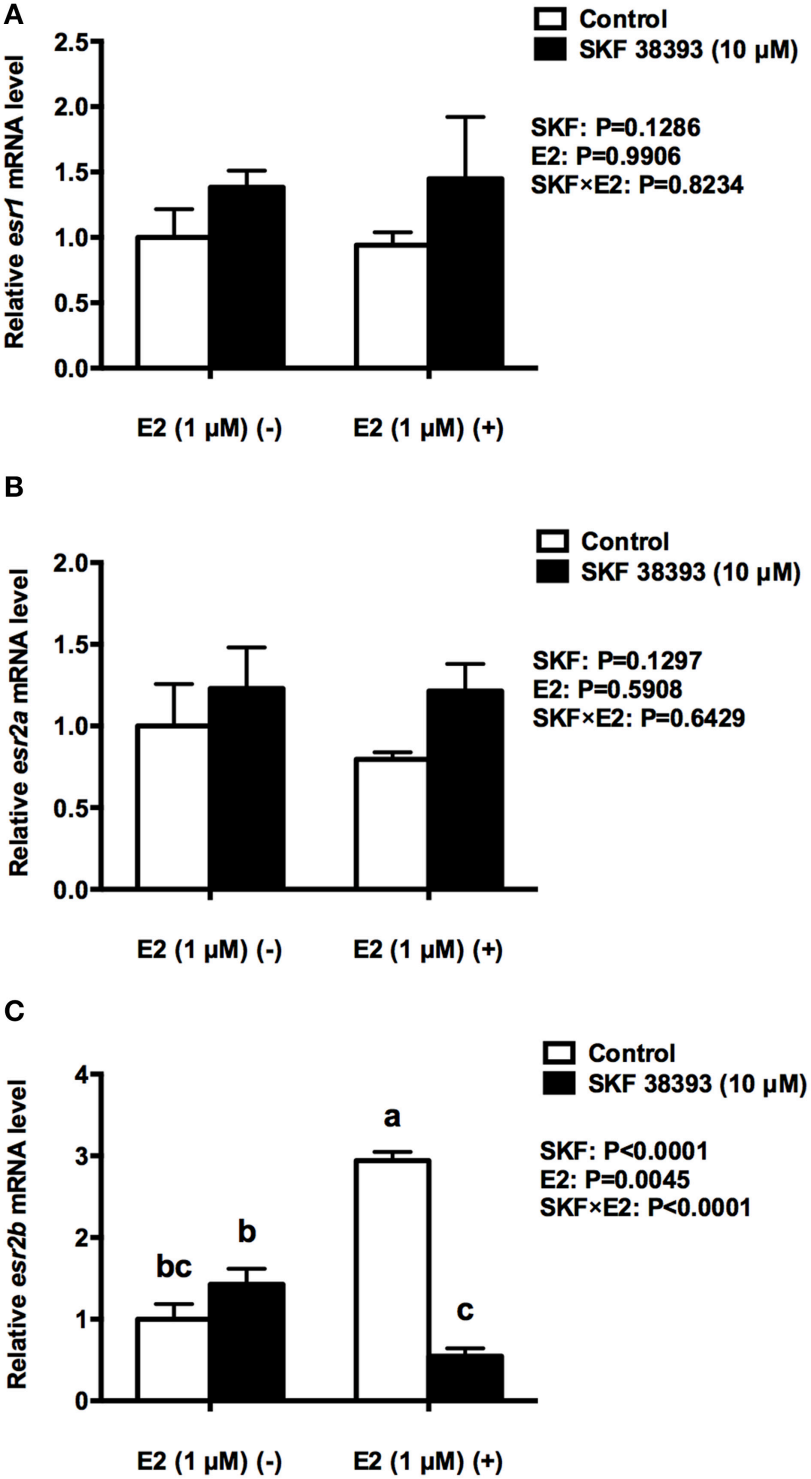
**Quantitative real-time PCR analysis showing variations in the relative amounts of the ***esr1*** (A), ***esr2a*** (B) and ***esr2b*** mRNA (C) in primary RGCs culture exposed to 1 μM E2 and 10 μM SKF 38393**. Data were normalized and defined as fold change relative to control, and bars represent the mean + SEM. Treatment groups marked by different letters are significantly different (*P* < 0.05).

Further analysis was essential in understanding the potential mechanisms of antagonistic regulation of *cyp19a1b* by the combined high concentration of E2 and high concentration of SKF 38393. As with the previous experiment, two-way ANOVAs were conducted that examined the potential interactive effects of SKF 38393 and E2 on *drd1a, esr1 esr2a*, and *esr2b* mRNA level and p-CREB protein level. Exposure to 10 μM SKF 38393 and 1 μM E2 alone did not affect *drd1a* (*F* = 0.9571, DFn = 1, DFd = 12, *P* = 0.3472) mRNA levels in cultured RGCs (Figure 6A). In contrast, there was a statistically significant interaction between SKF 38393 and E2 on p-CREB protein (*F* = 6.252, DFn = 1, DFd = 12, *P* = 0.0369) and *esr2b* mRNA (*F* = 86.01, DFn = 1, DFd = 12, *P* < 0.0001) levels. *Post-hoc* tests showed that higher concentration of E2 inhibited the up-regulation of p-CREB protein induced by SKF 38393 (*F* = 6.252, DFn = 1, DFd = 12, *P* = 0.0484; Figures 6B–D) and that SKF 38393 inhibited the up-regulation of *esr2b* mRNA induced by higher concentration of E2 (*F* = 86.01, DFn = 1, DFd = 12, *P* < 0.0001; Figure 7C) in RGCs, both following a similar pattern to that of *cyp19a1b* expression (Figure 3D). No effects on *esr1* and *esr2a* were evident (Figures 7A,B).

## Discussion

The findings of this study indicate that *cyp19a1b* expression is up-regulated in goldfish RGC cultures in response to individual treatments with E2 or D1R agonist. However, co-exposure to these substances leads to either a further up-regulation or a down-regulation of *cyp19a1b* mRNA levels that are dependent on the concentration of E2. This is the first evidence for direct multifactorial control of aromatase in RGCs from the adult teleost brain. In mammals, aromatase is expressed in both neurons and glia (Tsuruo et al., 1995; Azcoitia et al., 2003). In contrast, RGCs are the exclusive cells to express aromatase in teleost brains (Forlano et al., 2001; Tong et al., 2009). This makes teleosts amenable models to delineate direct control of glial aromatase expression. Our data provides the foundation for further analysis of neurotransmitter and estrogenic regulation of RGCs, and contributes to our understanding of neuronal-glial interactions. This is important because estrogens in the CNS control fundamental processes such as sexual behavior and neurogenesis (Okada et al., 2008; Ubuka and Tsutsui, 2014; Pellegrini et al., 2015).

The ability of E2 to regulate brain aromatase mRNA has been reported for a variety of species, including fish, birds, and rats (Hutchison and Steimer, 1986; Pellegrini et al., 2005; Zhao et al., 2007; Strobl-Mazzulla et al., 2008). Here, we confirm and extend these observations because we show a direct effects of E2 on isolated goldfish RGCs. Our pharmacological experiments using goldfish RGCs in culture indicate the involvement of ERs since the up-regulation of *cyp19a1b* caused by exogenous E2 was completely blocked by the ER antagonist MPP (Pinto et al., 2014), consistent with previous findings in zebrafish *in vivo* (Diotel et al., 2010). Here, we report that E2 *in vitro* can increase *esr2b* mRNA only at a relatively high concentration. Contrary to *in vivo* reports in teleost, we did not find effects of exogenous E2 on *esr1* and *esr2a* expression in RGC cultures (Menuet et al., 2005; Marlatt et al., 2008). To further understand the involvement of ERs, we administrated MPP together with SKF 38393 and E2. Exposure to MPP decreased the expression of all three nuclear ERs, suggesting that endogenous estrogen may be involved. Moreover, MPP blocked the stimulatory effect of added E2 on *cyp19a1b* expression. This finding indicates that the up-regulation of *cyp19a1b* mRNA by E2 is potentially due to the recruitment of the existing *esr1* and *esr2a* and/or increasing expression of *esr2b* to contribute to the positive feedback loop between aromatase, estrogens, and ERs (Diotel et al., 2010). Further studies on the involvement of *esr2b* are currently limited by the lack of a selective *esr2b* antagonist, although we do hypothesize that *esr2b* may be contributing to the up-regulation of *cyp19a1b* in RGCs, given the potency of *esr2b* to drive reporter gene expression for a consensus ERE construct or for the zebrafish *cyp19a1b* promoter construct in transfected cell assays (Menuet et al., 2002). It is unlikely that the G-protein coupled membrane estrogen receptor (GPER) is involved because we have been unable to identify GPER in RGCs using targeted PCR and RNA sequencing (data not shown).

We confirm here the results from our recent study (Xing et al., 2015) of the direct stimulatory effect of D1R activation of the p-CREB signaling pathway to increase the expression of *cyp19a1b* in adult female goldfish RGCs. Interestingly, in the current study, pretreatment with the estrogen antagonist MPP completely blocks SKF 38393-stimulated *cyp19a1b* expression. This suggests that DA does not increase the expression of ERs but somehow activates or recruits the pre-existing ERs that contribute to the increase in expression of *cyp19a1b*.

While it is clear from our data that treatments with E2 or SKF 38393 alone can increase expression of *cyp19a1b*, this is unlikely to represent situations *in vivo* where RGCs would be responding to neurotransmitters and endogenous estrogens simultaneously. Therefore, we treated RGCs with combinations of low and high concentrations of the D1R agonists SKF 38393 and low and high concentrations of E2. It is evident that the RGC *cyp19a1b* response to D1R activation is dependent on the level of E2. The maximal response in *cyp19a1b* expression (i.e., 8.0-fold) was obtained from RGCs exposed to the lower E2 level of 100 nM plus the concentration of 10 μM SKF 38393, which we showed previously to be the highest effective concentration in the culture system (Xing et al., 2015). These experiments revealed a synergistic interaction between E2 and SKF 38393 that was supported statistically. Additionally, the levels of cellular p-CREB most closely followed the pattern of *cyp19a1b* expression, however, a more complex effect on D1R and ER expression was evident. The low level E2 treatment appears to amplify signaling via D1R/cAMP/PKA/p-CREB. Our absolute quantification of *drd1a* mRNA abundance showed that the low concentration of E2 increased D1R gene expression, especially in the presence of the D1R agonist SKF 38393. With the increased expression level of D1R, we hypothesized the activity of the downstream signal transduction pathway will be higher than the control level. Our data showed that the protein level of p-CREB also increased upon exposure to E2 and SKF 38393, similar to the *drd1a* expression pattern. It is well-known that nuclear ERs bind to EREs thereby enhancing *cyp19a1b* transcription (Callard et al., 2001; Menuet et al., 2002, 2005) as transcription factors themselves or potentially through interactions with other transcription factors, such as activator protein 1 (Marino et al., 2006; Zhang and Trudeau, 2006). While our experiments with the ER antagonist MPP indicate that endogenous pre-existing ERs are involved, our findings do not support that an increase in ERs is required for the synergistic up-regulation of *cyp19a1b* by E2 and SKF 38393 in RGCs since no increase in expression was observed for any of the three nuclear ERs. Together these data indicate that the up-regulation of *drd1a* expression and p-CREB protein may contribute to the overall synergistic effects of the low concentration of E2 and SKF 38393 on *cyp19a1b* expression.

While we do not have an estimate of the intracellular or extracellular levels of estrogens at or near regions of high RGC density in the brain, we consider the high 1 μM concentration of E2 to be supraphysiological and the low 100 nM concentration to be physiological relative to circulating E2 (Kobayashi et al., 1988) based on previous *in vivo* responses to exogenous estrogens in goldfish (Martyniuk et al., 2006). Nevertheless, we originally hypothesized that the higher combined concentrations of E2 and SKF 38393 would cause up-regulation of *cyp19a1b* expression. However, co-treatment with 10 μM SKF 38393 significantly reduced the high *cyp19a1b* expression induced by 1 μM E2. This was evident at the level of intracellular signaling because p-CREB protein levels were also reduced by the co-treatment of high concentration of E2 and high concentration of SKF 38393. The effects of E2 on CREB are variable since both inhibition and stimulation have been reported, perhaps reflecting tissue-specific regulation (Duan et al., 1999; Choi et al., 2004). Our findings did not show any change in p-CREB protein levels after exposure to E2 alone regardless of the concentration. Moreover, 10 μM SKF 38393 reduced the increase in *esr2b* expression caused by the high 1 μM concentration of E2 in our RGCs. Even though SKF 38393 did not cause any down-regulation after 24 h exposure, time-course studies have shown that *esr2b* decreased after 8 h exposure to SKF 38393 alone in RGC culture (unpublished data). Although speculative, together these data indicate that the attenuation of p-CREB protein and *esr2b* expression may contribute to the overall antagonistic effects of the high concentrations of E2 and SKF 38393 on *cyp19a1b* expression.

**Figure 8:**
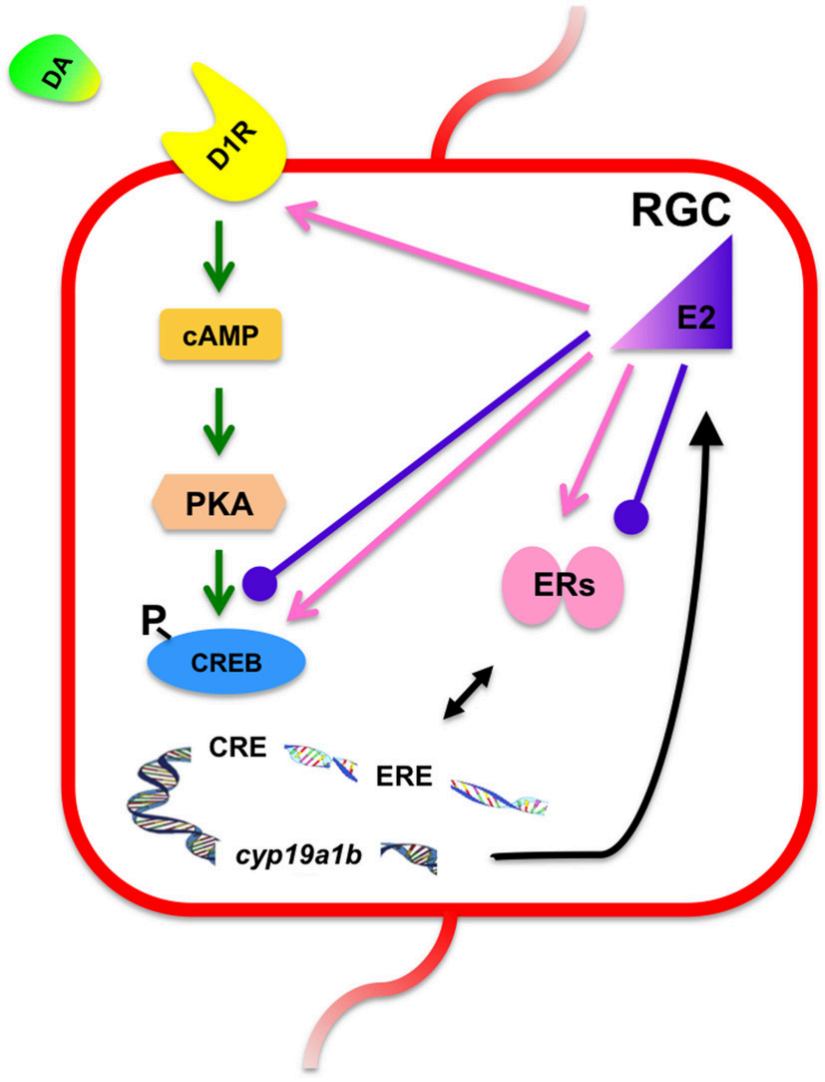
**Proposed mechanisms underlying the synergistic and antagonistic regulation of ***cyp19a1b*** expression in cultured RGCs**. Activation of the D1R by SKF 38393 (10 μM) up-regulates *cyp19a1b* expression, most likely through cAMP, PKA, and the phosphorylation of CREB. This up-regulation can be enhanced by low concentration of E2 (100 nM) through increasing the expression of D1R and the level of p-CREB protein (pink arrows). Also, up-regulation of *cyp19a1b* expression by E2 and DA both requires the involvement of *esr1* and *esr2a*. However, a high concentration of E2 (1 μM) and SKF38393 (10 μM) together failed to up-regulate *cyp19a1b* expression, potentially due to attenuation of *esr2b* expression and p-CREB levels (purple bulbous arrows). RGC, radial glial cells; DA, dopamine; D1R, dopamine D1 receptor; cAMP, cyclic adenosine monophosphate; PKA, protein kinase A; CREB, cAMP response element binding protein; p-CREB, phosphorylated cAMP response element binding protein; CRE, cAMP response element; E2, 17β-estradiol; ERs, estrogen receptors; ERE, estrogen response element.

In summary, we have described an example of complex control of aromatase B expression in adult teleost RGCs. Anatomical and physiological data (Xing et al., 2015) indicate a close relationship between catecholaminergic cell bodies and fibers and D1R-expressing RGCs in female goldfish brain. Pharmacological activation of D1R up-regulates *cyp19a1b* expression in RGCs, which ultimately would increase estrogen synthesis. It has been previously shown and confirmed here, that teleost RGCs respond positively to E2 with an increase in *cyp19a1b* expression. We extend these observations to demonstrate a potentiating or synergistic interaction between dopaminergic and estrogenic stimulation of *cyp19a1b* expression. On the other hand, it appears that overstimulation of RGCs with the combined high concentration of E2 and high concentration of SKF 38393 may limit *cyp19a1b* expression, and thus reduce estrogen synthesis in a manner similar to classical negative feedback control (Figure 8). In the teleost brain, the two main roles of estrogens in the adult brain that have been documented thus far are regulation of expression of neuroendocrine-related genes (Martyniuk et al., 2006), and the inhibition of neurogenesis (Diotel et al., 2013; Makantasi and Dermon, 2014). Interactive regulation of aromatase B by dopamine and estrogens therefore, would finely regulate local estrogen levels that in turn could regulate adjacent RGCs that express nuclear ERs, or neurons in the vicinity that express these receptors or GPER. However, further studies on the conversion of androgen precursors by aromatase B are needed to further understand the control of estrogen synthesis in the brain. Given the array of neurotransmitter and neuropeptide-expressing neurons and fibers in the neurogenic regions where *cyp19a1b* expressing RGCs are found, it is likely that many other neurohormones regulate the steroidogenic function of this critical cell. The culture system we have developed provides an amenable approach to the understanding of multifactorial control of RGCs.

## Author contributions

LX, designed the study, developed the methodology, conducted the majority of experiment, and analysis and wrote the manuscript. CE, maintained cell culture and helped with qPCR analysis. VT, helped with the design of the study and editing the article.

### Conflict of interest statement

The authors declare that the research was conducted in the absence of any commercial or financial relationships that could be construed as a potential conflict of interest.
